# Cross-species toxicogenomic analyses and phenotypic anchoring in response to groundwater low-level pollution

**DOI:** 10.1186/1471-2164-15-1067

**Published:** 2014-12-05

**Authors:** Immacolata Porreca, Fulvio D’Angelo, Daniela Gentilcore, Emanuele Carchia, Angela Amoresano, Andrea Affuso, Michele Ceccarelli, Pasquale De Luca, Libera Esposito, Francesco M Guadagno, Massimo Mallardo, Antonio Nardone, Sergio Maccarone, Francesca Pane, Marzia Scarfò, Paolo Sordino, Mario De Felice, Concetta Ambrosino

**Affiliations:** IRGS, Biogem, Via Camporeale, 83031 Ariano Irpino, Avellino, Italy; Department of Chemical Sciences, University of Naples Federico II, Via Cinthia, 80126 Naples, Italy; Department of Science and Technology, University of Sannio, via Port’Arsa 11, 82100 Benevento, Italy; Molecular Medicine and Medical Biotechnologies, University of Naples “Federico II”, Naples, Italy; Department of Public Health, University of Naples “Federico II”, Naples, Italy; Institute for Mediterranean and Forestal Systems (ISAFOM-CNR), Catania, Italy; Stazione Zoologica Anton Dohrn, Naples, Italy

**Keywords:** Microarray, Estrogen, Toxicology, Liver, Toxicogenomics, Low-dose, Chemical mixture, Groundwater pollution, Animal models

## Abstract

**Background:**

Comparison of toxicogenomic data facilitates the identification of deregulated gene patterns and maximizes health risk prediction in human.

**Results:**

Here, we performed phenotypic anchoring on the effects of acute exposure to low-grade polluted groundwater using mouse and zebrafish. Also, we evaluated two windows of chronic exposure in mouse, starting *in utero* and at the end of lactation. Bioinformatic analysis of livers microarray data showed that the number of deregulated biofunctions and pathways is higher after acute exposure, compared to the chronic one. It also revealed specific profiles of altered gene expression in all treatments, pointing to stress response/mitochondrial pathways as major players of environmental toxicity. Of note, dysfunction of steroid hormones was also predicted by bioinformatic analysis and verified in both models by traditional approaches, serum estrogens measurement and *vitellogenin* mRNA determination in mice and zebrafish, respectively.

**Conclusions:**

In our report, phenotypic anchoring in two vertebrate model organisms highlights the toxicity of low-grade pollution, with varying susceptibility based on exposure window. The overlay of zebrafish and mice deregulated pathways, more than single genes, is useful in risk identification from chemicals implicated in the observed effects.

**Electronic supplementary material:**

The online version of this article (doi:10.1186/1471-2164-15-1067) contains supplementary material, which is available to authorized users.

## Background

*In vitro* assays for compound toxicity are commonly based on the assumption that toxicants exposure results in changes in gene expression, a biological phenomenon predictive of successive morphological abnormalities [1–3]. Toxicogenomics, defined as changes in genome function that occur with toxicant interaction [4], is a sensitive, informative and measurable assay to complement traditional toxicological endpoints [5–7]. These advantages prompted the use of toxicogenomics to test the effect of single molecules or simple chemical mixtures [8, 9]. The objectives of transcriptomics in environmental studies (ecotoxicogenomics) are the accomplishment of classical toxicological and new molecular endpoints in the identification of exposure-related alterations, and proper consideration of the complex nature of anthropogenic pollution and bioaccumulation events [10–17]. Besides environments are frequently contaminated with multiple classes of compounds, only a limited number of toxicological studies have recently addressed this problem by using omics approaches to fish species, in environmental field [11, 18–20].

Ecotoxicogenomics is faced with determination of specific patterns of gene expression elicited by environmental samples with known or potential toxicity [12]. Transcriptome analysis has been successfully applied in testing low doses of environmental stressors in biological systems, thus leading to the identification of biomarkers that are easily detectable and related to the observed phenotype, the so called phenotypic anchoring [21, 22]. In this process, the integration of toxicogenomics data from different models is pivotal to validate deregulated patterns, to challenge the low signal to noise ratio and to predict potential risks for human health [23, 24]. Mouse and zebrafish studies indicate that gene expression profile approaches are successful in identifying chemical-specific patterns of altered gene expression [2, 25–27]; for this reason, and for their genetics and biology, these models are widely accepted by the scientific community for environmental toxicology studies [10, 28].

In populations living near waste dumpsites, the correlation between the exposure to chemical mixtures and health disorders has been monitored with different results [29–32]. Typically, low-level exposure to pollutant mixtures is frequently unappreciated and little is known about the consequences of chronic exposure in infants. Among people exposed to contaminants, infants and foetuses are thought to be more susceptible to insults from toxic chemicals because of the period of rapid development [33, 34]. This is an important issue since the adverse effects of a long-term corollary of foetal/neonatal exposure to different pollutants can remain undetected till diseases develop in the adulthood. Several studies have investigated the leachate composition [35–37] and related cytotoxicity/mutagenicity in eukaryotic systems, suggesting the potential of leachate to cause harmful effects to public health through seepage into groundwater. Poorly concentrated pollutants remain undetected while they are transformed and enter the food chain. Moreover, their toxicity is underestimated if cocktail effect and bioaccumulation over long-term exposure is not considered.

In the present study, we investigate the effects of exposure to environmental low-level polluted water for distinct exposure time and developmental windows, with a focus on liver toxicity in two model systems, mouse and zebrafish. Methodologically, we correlate microarray data with phenotypic and chemical parameters after short-term exposure of mice and zebrafish, and long-term exposure of mice, to environmental low-grade polluted water. Our findings are a proof that toxicogenomics applied to environmental toxicology studies permits new biomarker identification and risk assessment in the common situation of low-grade pollution and different exposure timing.

## Results

### Toxicogenomic evaluation of "acute" exposure in mice

To investigate the effects of exposure to environmental polluted waters, samples were collected from dumps located upstream (U) and downstream (D) a sanitary landfill, both insist on the same aquifer. Analyses aimed at the definition of aromatic and heavy metal contents in water samples were carried out by GC-MS and ICP-MS techniques. Additional file 1 reports results representative of different samplings, underlining the similarity between U and D waters compared with control water.

The impact of acute exposure to sampled groundwater was investigated by treating 21 PND CD1 mice for 3 months. Outbred CD1 mice were chosen to avoid the influence of genetic background on any phenotypic aspect. During the treatment, no differences were recorded in water/food consumption as well as in mortality and body weight between the two treatment groups compared with control (data not shown).

**Figure 1:**
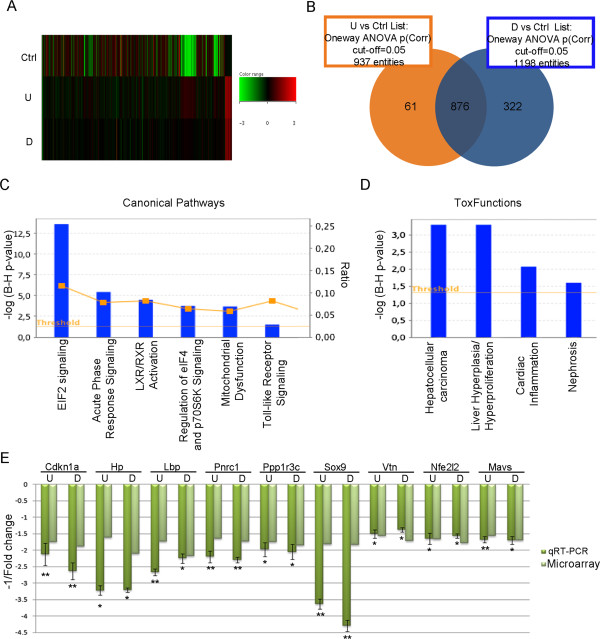
**Molecular characterization of acutely exposed mice liver. (A)** Heatmap showing the expression profiles in Ctrl, U and D waters treated mice livers. The expression value of each gene is mapped to a colour-intensity value, as indicated by the colour bar. **(B)** Venn Diagram showing the probe sets overlap in U (orange) and D (blue) groups compared to the control. **(C)** Canonical pathways, identified by IPA analysis, deregulated in liver of U and D waters exposed mice. The left *y*-axis value is the negative lg_10_(Benjamini-Hochberg corrected *p*-value). The orange squares referred to the right *y*-axis represent the ratio values indicating the percentage of genes in the pathway that are also deregulated. **(D)** Toxfunctions deregulated in U and D exposed animal livers. **(E)** qRT-PCR validation of some DEGs. Data are reported as the negative inverse of fold change value calculated as ratio between average expression in U/D and in Ctrl exposed animals (8 animals/group).

**Figure 2:**
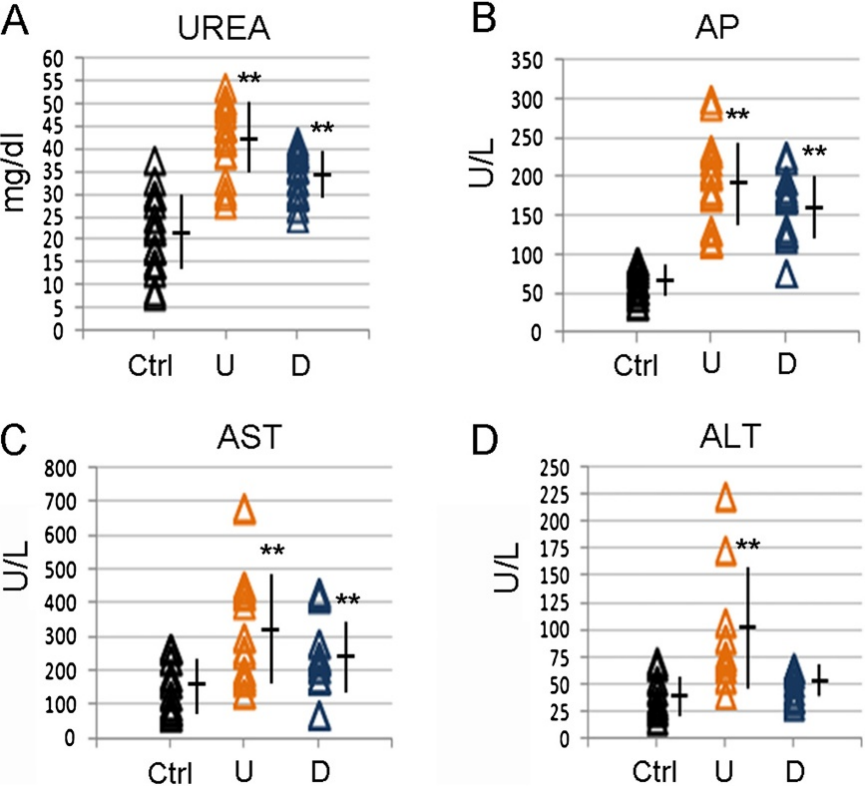
**Biochemical analyses in acutely exposed mice. (A)** Serum Urea, **(B)** AP, **(C)** AST and **(D)** ALT levels were measured in Ctrl (black), U (orange) and D waters (blue) treated mice (20 animals/group). Each sign is a single mouse. Mean and standard deviation is reported. **p-value ≤0,01.

Since the liver is highly sensitive to toxicant exposure, gene expression profiling analysis of the "acute response" was performed on RNAs obtained from 9 livers/group and using Affymetrix mouse whole genome. Different probe sets were retrieved in U- and D-treated mice, as shown in Volcano Plots and related tables (Additional file 2). The Heatmap and the Venn Diagram highlight many common and unique Differentially Expressed Genes highlight many common and unique Differentially Expressed Genes (DEGs) in U and D group mice (Figure 1A, B). The observation of U- and D-specific DEGs suggests that these waters were similar but not identical (Additional file 1). Common DEGs were further investigated as true markers of groundwater exposure. Using the Ingenuity Pathway Analysis program (IPA, see Materials and Methods section), we identified several biofunctions relevant to the data set, including Hepatic System Disease (*p-*value = 2,73E-03) and Renal and Urological Disease (p-value = 1,37E-03). The IPA analysis of canonical pathways suggested that transcriptomics-based evidence of alterations in liver activity may be related to perturbation of pathways involved in liver stress responses (Eif2 signalling), or/and to mitochondrial dysfunction (Figure 1C). In the deregulated mitochondrial pathway, we found alteration of several genes involved in the respiratory chain, mostly codifying for components of the NADH:ubiquinone oxidoreductase (complex I, Ndufaf1, Ndufs5, Ndufab1, Ndufb6, Ndufa3). The liver alteration is also supported by toxfunction prediction performed within the IPA analysis (Figure 1D). qRT-PCR analysis of genes related to the two top impinged toxfunctions confirmed microarray data and other IPA tools bioinformatics observations (Figure 1E).We analysed hepatic and renal activity in acutely treated mice by measuring serum alanine and aspartate transaminases (ALT, AST), alkaline phosphatase (AP), and urea levels. Values of urea, AP and AST were significantly increased in U and D groups (Figure 2A-C), in agreement with bioinformatics prediction of liver suffering. Conversely, ALT levels were higher only in U animals (Figure 2D). However, hematoxylin/eosin staining of liver sections showed no macroscopic signs of tissue alteration (data not shown).

### Comparing mouse and zebrafish transcriptomes

The identification of deregulated genes in common between mammals and fish may elicit the identification of reliable biomarkers of environmental exposure. Due to zebrafish susceptibility to groundwater treatment, 3 months old fish were enrolled in each treatment group. After 3 months exposure, the treatment was stopped to avoid suffering since animal weight and length were reduced relative to control (Additional file 3).

**Figure 3:**
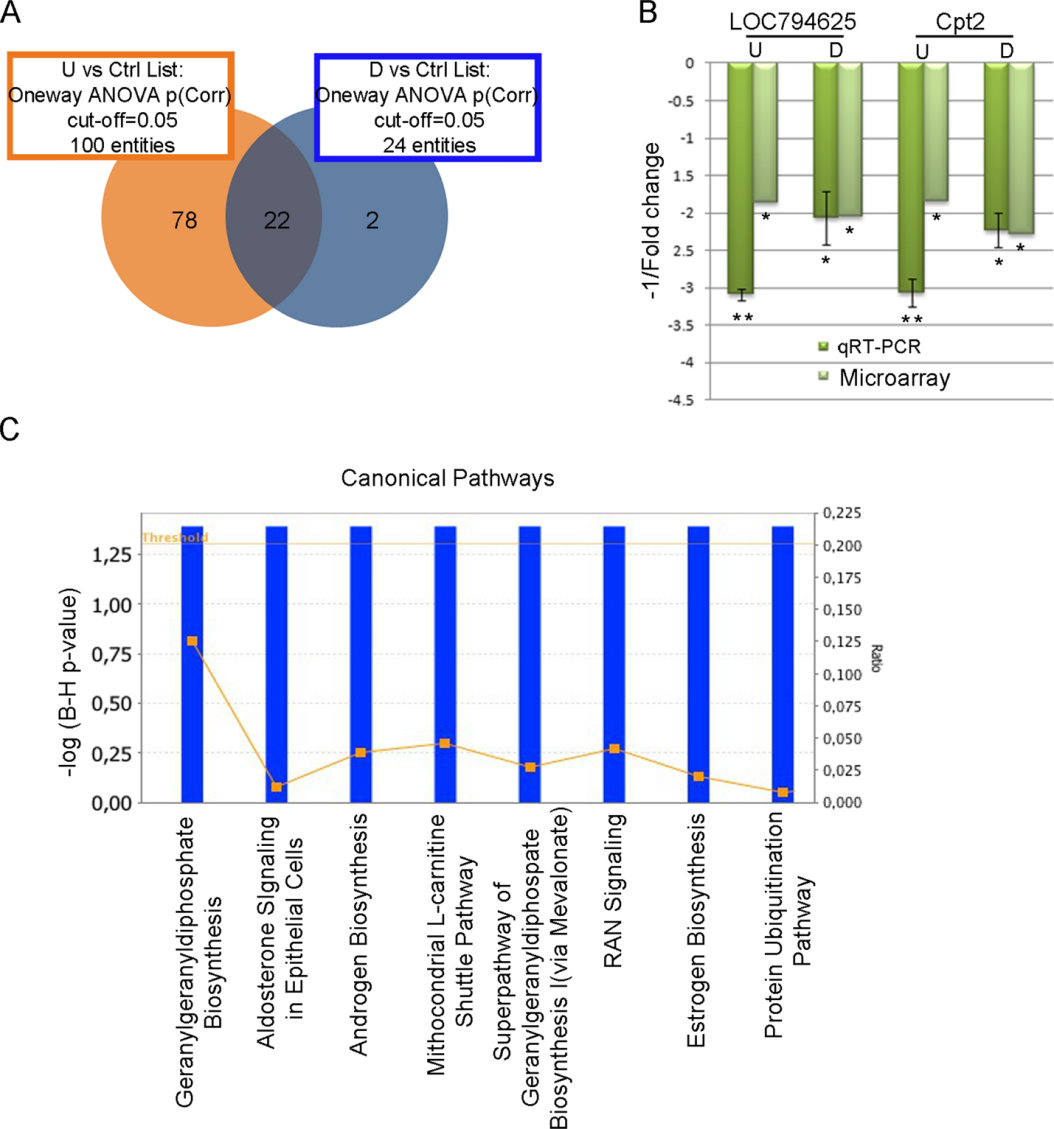
**Toxicogenomics analyses in acute exposed zebrafish. (A)** Venn Diagram showing the probe set overlap in U (orange) and D (blue) compared to the control group. **(B)** qRT-PCR validation of selected genes (*LOC794625* and *Cpt2*) deregulated in exposed zebrafish livers. Data are reported as the negative inverse of fold change value calculated as ratio between average expression in U/D and in Ctrl exposed animals (8 animals/group). **(C)** Canonical pathways identified by IPA analysis on common DEGs in zebrafish livers. See Figure 5 caption for graphic description.

Transcriptome analysis revealed more changes in gene expression in fish treated with D water (100 genes, 86 down-regulated) than in those exposed to U water (24, 20). Almost all U-specific altered probes were included in the D list (Figure 3A). Volcano plots for these experiments are reported in Additional file 3. Validation by qRT-PCR of two common DEGs is showed in Figure 3B. Gene Ontology analysis has been performed to compare the two acute exposure profilings in mice and zebrafish. 81 gene ontology terms were significantly deregulated in mice with a p-value of 0.05 (Additional file 4). In zebrafish only 3 gene ontology terms were significantly deregulated (Additional file 5). Among them the mitochondrion was shared by the two profilings. Among the 3 genes enrinched in GO mitochondrion term in zebrafish, we validated the reduction of *ctp2* transcript, codifying for a protein involved in the β-oxidation of long-chain fatty acids in the mitochondrial inner membrane. Besides the unchanged expression of respiratory chain genes, lower *ctp2* levels can result in a decrease in the production of ATP. Reduced *cpt2*expression, a PPARα-target gene, was implicated in the development of hepatic steatosis and toxicity in the livers of both zebrafish exposed to the environmental contaminant perfluorononanoic acid and high fat diet-fed mice treated with PCB 15 [38, 39]. IPA analysis of common DEGs highlighted steroid hormone biosynthesis among deregulated canonical pathways (Figure 3C) but could not predict any liver function alteration (data not shown).

**Figure 4:**
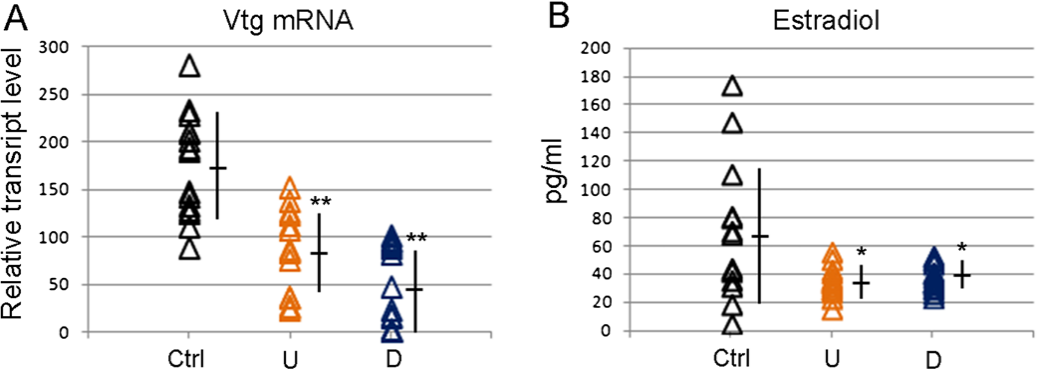
**Validation of hormonal alteration in acutely exposed mice and zebrafish. (A)** *Vtg* mRNA level measured by qRT-PCR in liver of zebrafish females (20 animals/group). Each sign is a single animal. **(B)** Serum estradiol levels measured by ELISA assay in acute exposed mouse females (15 animals/group). Each sign is a single animal. Mean and standard deviation is reported. *p-value ≤0,05, **p-value ≤0,01.

Comparison of data sets from acutely exposed mouse and zebrafish revealed two molecular responses in common, *i.e.* the altered activity of steroid hormones synthesis/signalling biofunctions, and the prediction of aromatase (Cyp19A) as upstream regulator (*p*-value = 4,95E-05 in mouse, *p*-value = 4,68E-05 in zebrafish), in agreement with the detection of trace chemicals affecting fertility [40]. To verify IPA prediction, estradiol synthesis was examined in both models. Expression of the *vitellogenin* (*vtg*) gene, typically estrogen-dependent, was reduced in treated zebrafish, in line with estrogen findings. Likewise, plasmatic estradiol levels in acutely exposed mice were in line with IPA-based prediction (Figure 4B). Thus, data suggest that the comparison of toxicogenomics data sets in zebrafish and mouse allows recognition of altered biofunctions.

### Toxicogenomic evaluation of chronic exposure started in utero or at 21 PND

**Figure 5:**
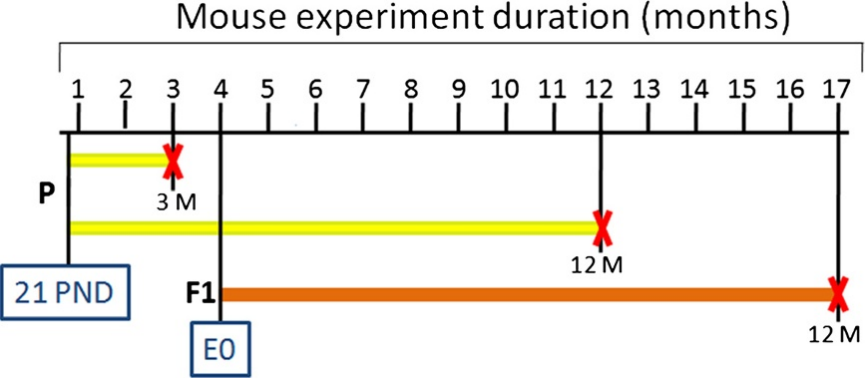
**Schematic representation of exposure windows in mice.** P generation mice were treated for 3 (acute exposure) and for 12 months (chronic exposure), starting from 21 PND. F1 generation mice, obtained by crossing 4 months old P mice, were treated for 12 months starting from embryonic day 0. In black horizontal line the experiment duration; in yellow and orange exposure duration for P and F1, respectively; the red crosses indicate the end of the treatment.

**Figure 6:**
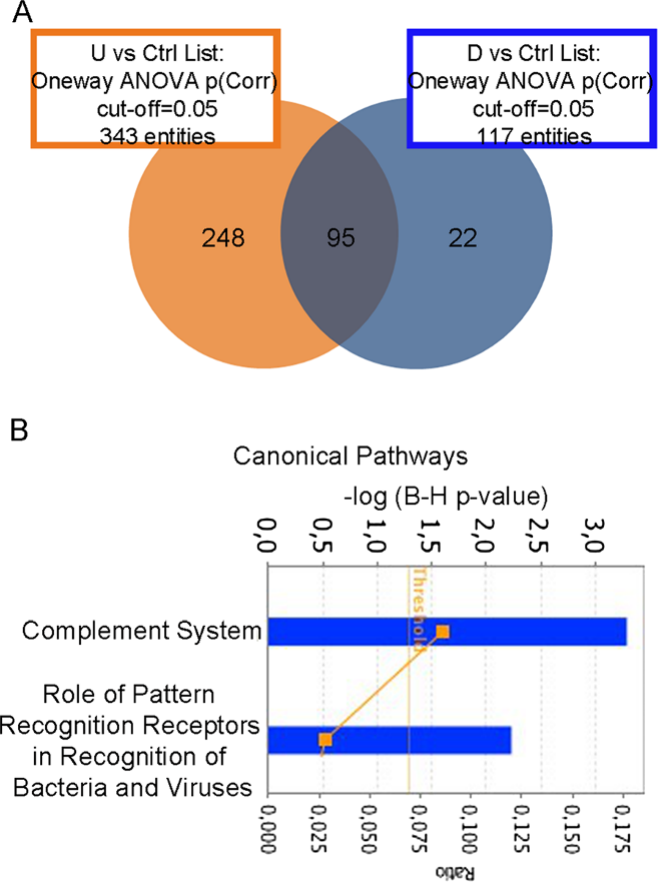
**Transcriptomics analyses in livers of 12 months P exposed mice. (A)** Venn Diagram showing probe set overlap between U (orange) and D (blue) compared to the control group after 12 months of treatment started at PND 21. **(B)** Canonical pathways deregulated in liver of exposed P mice. See Figure 5 caption for graphic description.

**Figure 7:**
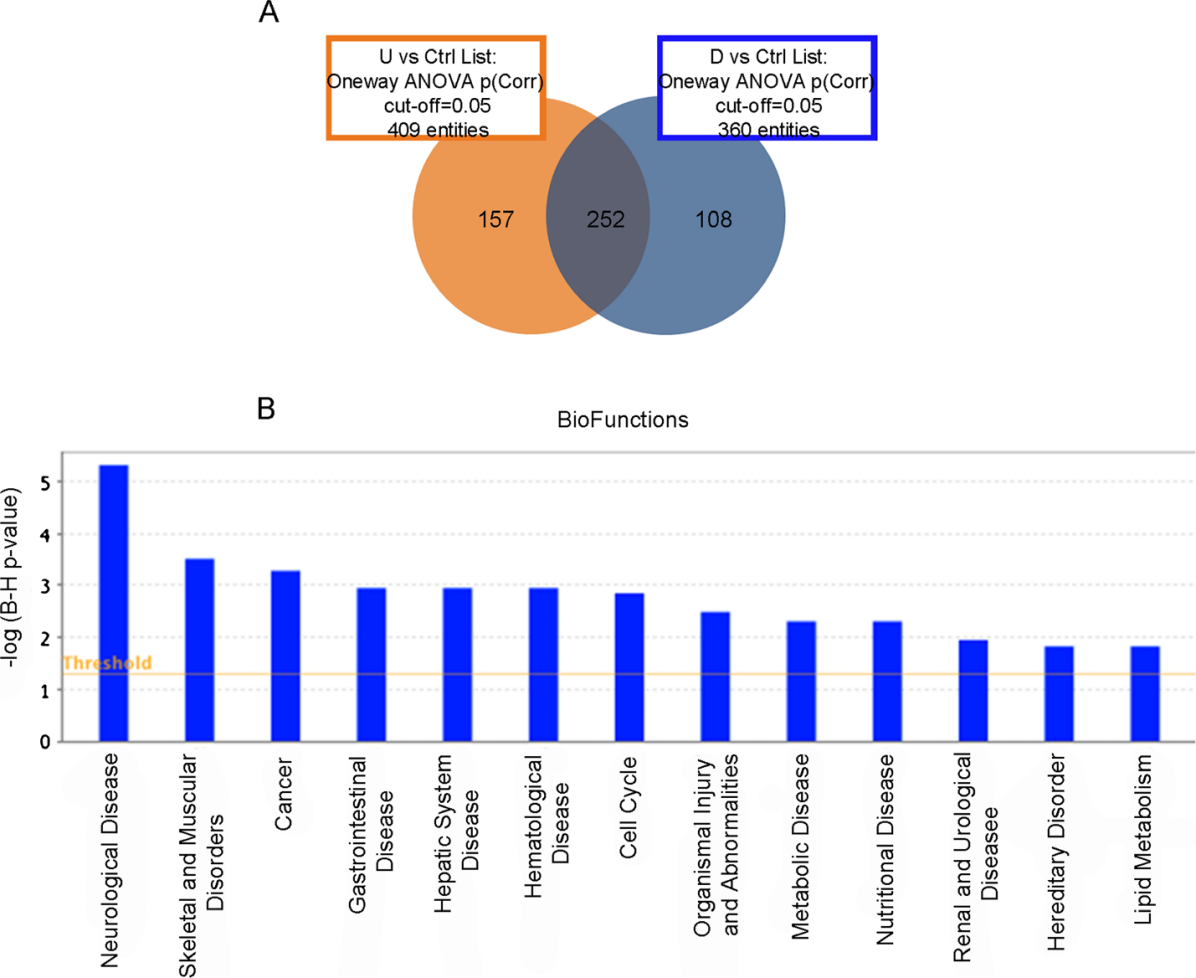
**Transcriptomics analyses in livers of F1 mice exposed in utero and for 12 months. (A)** Venn Diagram showing the probe set overlap between U (orange) and D (blue) compared to the control group after 12 months of treatment started at GD 0 in F1. **(B)** Biofunctions deregulated in liver of F1 exposed animals. See Figure 5 caption for graphic description.

In order to analyse the effects of chronic exposure (12 months) under different exposure windows, mice treatment started at 21 PND or *in utero* (F1 from four months old exposed CD1 mice) (Figure 5).Transcriptome analyses conducted on livers at the end of treatment showed extreme reduction of deregulated probes in chronic exposed parents (P) (Figure 6A) and offspring (F1) (Figure 7A) compared to the acute treatment. This reduction could be interpreted as indication of a compensation effect in chronically exposed mice.

Probe sets deregulated in P mice watered with U and D water shared several DEGs (Figure 6A). Furthermore, IPA analysis recognized numerous biofunctions related to impinged liver function (*e.g.* delay in initiation of liver repair; data not shown). Two canonical pathways, involving the complement system, were significantly altered (Figure 6B). They were not highlighted by acute exposure data, suggesting the specificity of chronic exposure effects. For this reason, elements of these complement system canonical pathways were used to validate microarray data by qRT-PCR (Additional file 6).

Bioinformatics analysis indicated seleno-methyl-selenocysteine (*p*-value = 7.12E-04) and phenol derivative (*p*-value = 3.44E-03) as upstream regulators of chemical toxicity. Remarkably, traces of selenium and phenolic compounds were retrieved in the sampled waters (Additional file 1). Analysis by toxfunctions (liver damage, *p*-value = 3.7E-02; liver cholestasis, *p*-value = 3.7E-02; liver necrosis/cell death, *p*-value = 3.7E-02) evidenced predictable signs of hepatic toxicity (Additional file 6), prompting us to deepen the assessment of liver/renal functionality in P mice by assaying urea, AP, ALT and AST serum levels. We found that levels of urea and AP were significantly increased in the treated mice versus control (Additional file 6). Histopathology evidenced the alteration of hepatic tissue, with the presence of typical signs of micro-vescicular steatosis in treated mice (Additional file 6).A high number of U/D-shared deregulated probes was observed also in chronically treated F1animals (Figure 7A). IPA analysis of common DEGs identified many biofunctions related to cell cycle alteration and cancer (Figure 7B) that were not detected in the P mice analysis. Thus, they could be considered related to the foetal exposure.

In order to identify exposure-specific signatures, we looked for common DEGs deregulated by U and D treatment in both chronic and acute exposure conditions in mice. Among 30 DEGs common to both data sets, the majority codifies for mitochondrial tRNA (Table 1), in agreement with mitochondrial alteration in liver stressing conditions [41]. 15 out of the 22 mitochondrial tRNAs were strongly up-regulated in both acute and chronic exposure; the reason for their increase is not clear. However it has been reported that hypoxia induces a similar effect in cardiomyocytes [42].

**Table 1:** **Genes deregulated in livers of both 3 and 12 months mice exposed to U and D waters**

Transcripts Cluster Id	Gene name	Gene description	Fold change			
			3 months		12 months	
			U vs Ctrl	D vs Ctrl	U vs Ctrl	D vs Ctrl
10603833	Usmg5	Upregulated during skeletal muscle growth 5	-4.53	-4.28	-2.83	-2.95
10374476	Rps17	Ribosomal protein S17	-2.96	-3.19	-1.85	-1.89
10357242	Dbi	Diazepam binding inhibitor	-2.87	-2.71	-1.84	-2.23
10387816	Rnasek	Ribonuclease, RNase K	-2.17	-2.33	-1.61	-1.78
10344817	Cspp1	Centrosome and spindle pole associated protein 1	-1.75	-1.62	-1.80	-2.38
10483809	Nfe2l2	Nuclear factor, erythroid derived 2, like 2	-1.66	-1.78	-1.71	-1.81
10500798			-1.57	-1.78	-2.75	-2.78
10598089	mt-Te	tRNA glutamic acid, mitochondrial	2.12	2.47	4.20	5.01
10598087	ND6	NADH dehydrogenase subunit 6	2.82	3.37	1.86	3.73
10598073	mt-Tq	tRNA glutamine, mitochondrial	4.07	5.85	5.67	7.28
10528191	Speer4d|Speer4c|Speer4e|	Spermatogenesis associated glutamate (E)-rich protein 4d/4c/4e	4.13	4.67	1.65	1.80
10598083	mt-Ts1	tRNA serine 1, mitochondrial	5.32	6.71	3.00	4.83
10598091	mt-Tp	tRNA proline, mitochondrial	5.62	7.62	3.11	3.85
10598075	mt-Ta	tRNA alanine, mitochondrial	5.93	7.82	16.92	26.75
10598023	mt-Tv	tRNA valine, mitochondrial	6.17	6.69	4.21	5.75
10519811	Speer8-ps1|Speer7-ps1	Spermatogenesis associated glutamate (E)-rich protein 7/8, pseudogene 1	6.34	6.92	3.13	3.88
10598057	mt-Tr	tRNA arginine, mitochondrial	6.40	7.63	10.12	28.13
10436773	Gm7735|Gm9789		6.48	6.90	3.75	3.25
10412517	Gm3002|Gm10021|Gm3512		7.66	10.80	3.88	4.55
10582888			7.70	9.65	2.83	5.64
10598081	mt-Ty	tRNA tyrosine, mitochondrial	10.46	13.91	6.51	10.49
10598079	mt-Tc	tRNA cysteine, mitochondrial	10.88	15.64	4.72	7.30
10598018	mt-Tf	tRNA phenylalanine, mitochondrial	11.56	14.23	14.71	27.37
10598062	mt-Th	tRNA histidine, mitochondrial	13.20	15.78	27.68	46.30
10598077	mt-Tn	tRNA asparagine, mitochondrial	21.72	32.04	6.55	9.85
10598041	mt-Tk	tRNA lysine, mitochondrial	24.14	28.86	39.43	66.79
10598071	mt-Tt	tRNA threonine, mitochondrial	27.51	35.77	7.81	15.37
10598064	mt-Tl2	tRNA leucine 2, mitochondrial	58.00	70.56	44.83	70.91
**10353034**	**Snord87**	Small nucleolar RNA, C/D box 87	-3.20	-3.39	2.83	2.77
**10398451**	**Rps25|Gm4963**	Ribosomal protein S25 | predicted gene 4963	-1.70	-1.81	1.75	1.80
**10465831**	**5730408K05Rik**	RIKEN cDNA 5730408 K05 gene	-2.26	-2.30	4.41	4.10

In bold, genes inversely deregulated in the two data sets. mt- stays for mitochondrial.

The arithmetic fold change is reported.

To identify hallmarks of groundwater chronic toxicity, we looked for genes deregulated in both P and F1 upon 12 months treatment. As described in Material and Methods and depicted in Figure 5, P and F1 animals received the same waters for most of the treatment, the only difference being the exposure window. However, only 9 deregulated probes were present in both lists, of which only two DEGs were similarly regulated (*Usmg5*, *Nd6*) (Table 2). The reported data suggest that the effects of acute *vs* chronic exposures as well as the effects of chronic exposure windows were mainly specific, likely as a consequence of adaptive response.

**Table 2:** **Genes deregulated in chronic exposure liver tissue of U and D treated mice in both P and F1**

Transcripts Cluster Id	Gene name	Gene description	Fold change			
			P		F1	
			U vs Ctrl	D vs Ctrl	U vs Ctrl	D vs Ctrl
10572813	Usmg5	Upregulated during skeletal muscle growth 5	-2.89	-3.19	-1.54	-1.66
10598062	NC_005089*		27.68	46.30	5.28	3.63
10598064	NC_005089*		44.83	70.91	3.40	2.29
10598087	ND6	NADH dehydrogenase subunit 6	1.86	3.73	3.76	4.43
**10448182**	**Mir703**	microRNA 703	1.61	1.64	-1.74	-1.73
**10507870**			3.51	3.20	-1.76	-1.94
**10507872**			3.59	3.32	-1.86	-2.16
**10512487**	**Rmrp**	RNA component of mitochondrial RNAase P	2.49	2.66	-2.10	-2.30
**10516908**	**Snora73a**		4.51	4.26	-2.36	-2.49

*Mitochondrial genome.

In bold, genes inversely deregulated in the two data sets parents (P) and offspring (F1).

The arithmetic fold change is reported.

## Discussion

Toxicogenomics aims to identify genes whose altered expression is associated to observed phenotypes or unpredicted outcomes. Matching high-throughput transcriptional approaches to traditional toxicological criteria is considered a powerful method for testing the impact of low-dose pollutant mixtures, particularly in natural settings. Here, we evaluated low-level contaminated groundwater toxicity by both approaches in vertebrate mouse and zebrafish. Determination of polluted groundwater toxicity in the experimentally controlled animal facility reduces disturbing factors encountered with environmental sampling of wild organisms.

Bioinformatics analyses of liver transcriptomics profiles in the acute exposure treatments revealed the effect of pollutants, whose presence in groundwater was supported by chemical testing (*e.g.* dibutyl phthalate, *p-*value = 1.46E-4) (Additional file 1). The alteration of pathways involved in stress response (EIF2-, eIF4-, p70S6K- signalling pathways) suggested their relevance in the evaluation of environmental exposure. Phenotypic and molecular data obtained in the analysis of acutely exposed mice point at liver toxicity, and advise a particular role for the stress response and mitochondria related pathways in its development. Indeed, the IPA analysis on acutely treated mice highlighted, among the canonical pathways, the mitochondrial dysfunction including genes for the mitochondrial respiratory chain (Ndufaf1, Cox6b10, Ndufs5, Cox6a1, Uqcr11, Ndufab1, Ndufb6, Ndufa3). All of them are nuclear genes down-regulated in the liver of acutely exposed mice. This condition could lead to the impairment in the electron transport and ATP synthesis and, overall, could compromise the health of liver cells. Inhibition of the respiratory and impairment of complexes I as well as of mitochondrial β-oxidation has been frequently associated to acute exposure to drugs and hepatocytes toxicity [43]. Furthermore, down-regulation of electron transport complex genes as well as mitochondrial alterations has been associated with several diseases, being chronic liver disease among them [44–49].

Among the genes whose expression level was altered in the acutely exposed mice, we found downregulation of *Cdkn1a* and *Nfe2l2. Cdkn1a* is the gene codifying for p21, which is a p53-dependent key regulator of cell fate, as it triggers cell cycle arrest in the G1 phase under various stress conditions [50]. It has been recently suggested that reduced p21 expression can support liver fibrosis through reduced hepatic stellate cells senescence [51]. It is relevant to note that in our experimental condition, hepatic stellate cells activation was one of the disturbed canonical pathways (*p*-value = 4,3E-001). As stated in the Results section, *Nfe2l2* is one of the few genes deregulated also in the chronic exposed mice (Table 1), where we found evidence of micro-vescicular steatosis (Additional file 6). The transcription factor Nfe2l2 plays a protective role in hepatic cells as a key regulator for induction of detoxifying enzymes, antioxidative stress genes and several other enzymes involved in cellular protection. Its deficiency leads to exacerbation of chemical hepatotoxicity and to a considerable increase in micro and macro-vescicular steatosis [52].

Indeed, chronically exposed mice showed progressive alteration of liver parenchyma and increased level of alkaline phosphatase, as reported in human non-alcoholic steatohepatitis where it often evolves in hepatocellular carcinoma, a disease pathway whose alteration was seen in the present research. A compensation effect was evidenced at molecular level by the reduction of DEGs. Sets of deregulated biofunctions were almost overlapping in acute and chronic exposure (data not shown). One exception was the specific alteration of the complement system. C1qa, c1qb and c1qc are members of the complement cascade, a fundamental component in the innate immunity helping in the clearance of pathogens. Recent studies demonstrated that complement activation may contribute to cancer growth by facilitating the dysregulation of mitogenic signaling pathways [53]. The three complement system genes were up-regulated in the liver of chronically exposed mice; in particular, overexpression of *C1q* complement system components may be envisaged as indicative of hetapotoxicity since its transcription in liver hematopoietic cells was induced in several types of damage [54, 55].

The possibility that exposure during foetal life could be more harmful than in the adulthood has been explored [33, 34, 56]. Here, our results confirm the selective impact of the exposure window in DEG changes, as only few genes (*Usmg5* and *Nd6*) were shared among parents and offspring. *Usmg5* was first recognized as a gene whose mRNA level increased during skeletal muscle growth in rats [57]. USMG5 protein was shown to be associated with ATP synthase in the mitochondria [58] and to contribute to the maintenance of ATP synthase population, an indication of its importance in cellular energy metabolism [59]. ND6 is one of the 7 mitochondrial DNA (mtDNA) encoded subunits of respiratory Complex I, again suggesting the alteration of mitochondria biology in the exposed animals also in chronic exposure conditions.

DEGs involved in cancer development (cell cycle and DNA repair) were specifically observed in offspring. Even if cancers were not observed in the collected organs, genotype context or longer exposure time could play a major role in their development. The identified genes could represent a predictive biomarker of exposure and effects whose identification is pivotal in monitoring. Interestingly, increased incidence of cancers has been reported in people leaving in landfill areas, where they are potentially exposed to polluted groundwater used for agriculture or other human activities. Further work is necessary to explore this hypothesis.

In toxicogenomics, studies of gene expression alteration in phylogenetically distant species are supposed to assist in the identification of altered pathways that impinge on human health. To this aim, we investigated the effects of polluted groundwater exposure on mice and zebrafish liver by toxicogenomics. Even if no common DEGs were retrieved in both vertebrates, bioinformatics analysis and phenotypic assays indicate aromatase and estrogen biosynthesis changes. These findings agreed with the suggestion that functional pathways could be better and reliable markers than single genes. This observation is consistent with the documented effect of several water pollutants (metals, plasticizers and others) on hormonal axes linked to reproductive health outcomes [60–64], as seen also in our experimental settings (manuscript in preparation). Here, toxicogenomics showed its predictive strength in identifying biomarkers, steroid hormones, that can be easily detected in blood, suitable for monitoring the impact of anthropogenic activities on human and identifying risks for human populations even if not indicative of specific pollutants.

In all treatment conditions, observed phenotypic effects can correlate to alterations in the stress response pathways and, mainly, in mitochondria activities, as supported by the ability of leachates to induce oxidative stress in organs [65, 66]. These pathways could be effectors of low-dose mixture toxicity, playing a role in the alteration of liver function and in the regulation of hormones biosynthesis, as previously shown [14].

## Conclusions

The reported data suggest that toxicogenomics analyses of different animal models and exposure conditions (timing and window) have the potential to disclose unpredicted outcomes and, most importantly, pathways useful for human and environmental health risk assessment also in conditions of low-level exposure. Our study points to the importance of considering pathways more than single gene alteration in toxicological assessment. It also allows the identification of specific pathways, with mitochondria as key factors of toxicity response, and sexual hormones as biomarker in environmental assessment.

## Methods

### Geochemical analyses

Water sampling was performed in a landfill located in southern Italy (Lat 41° 12′ 23,54′ ‘N; Long 15° 12′ 27,63′ ‘E, datum WGS84), with the permission of the municipal local authority. The present study did not involve endangered or protected species.

Water samples were collected from five 15 meters deep piezometers that are located in the saturated portion of an aquifer consisting of poorly permeable lithologies. Piezometers are situated transverse to the axis of groundwater flow: two upstream and three downstream the landfill (referring to the water flow direction). The sampling occurred every 15 days under dynamic conditions by means of a micro pump.

Aliquots of water solution (1 ml) from each sample were directly analysed by Inductively Coupled Plasma Mass Spectometry (ICP-MS) with an Agilent 7700 ICP-MS (Agilent Technologies), as described in detail in Additional file 1. Upon liquid-liquid extraction with chloroform, hexane and dichloromethane (1:1 v/v, performed 3 times), organic substances were collected, dried and dissolved in 200 μl of hexane, before GC-MS analysis performed on a 5390 MSD quadrupole mass spectrometer (Agilent Technologies), as detailed in Additional file 7.

### Animals and treatments

Groundwater used for animal treatment was filtered through glass and 0.22 μm (Millipore) filters and stored at 4°C.

Animal experiments were performed in compliance with the European Council Directive 86/609/EEC and the Italian Legislation on Animal Experimentation (D.Lvo 116/92) and procedures were approved (ID number 21-2009) by the Ethical committee named CESA (Committee for the Ethics of the Experimentations on Animals) of the Biogem Institute of Genetics Research "Gaetano Salvatore" (IRGS). The project has been communicated to the employed office of the Ministry of Health following the rules of the D.Lvo 116/92.

Mice were kept under standard facility conditions (22 ± 2°C, 55 + 10% humidity, 12:12 h light-dark cycle) in a specific pathogen-free facility. Animals received water and standard diet (4RF21 form Mucedola) "*ad libitum*"; type II EU cages, in polysulfone, had space to allow motility and parental care. Mice were sacrificed by carbon dioxide inhalation.Fifty 21 Post Natal Days (PND) CD1 mice (outbred, 30 male and 30 female) were randomly recruited for each of the three F0 treatment groups that received drinking tap-water (control, Ctrl), waters sampled from dumps upstream (U) or downstream (D) the landfill. Sampling from two different locations around the landfill area were undertaken to perform a robust toxicogenomic analyses, based on different (up and down) data sets whose shared elements were used for bioinformatics analyses. We chose this approach in order to eliminate from the analyses genes whose deregulation could be related to other reasons (noise). After 3 months of treatment (acute exposure, P generation), twenty mice/group were sacrificed, blood and several organs collected. Remaining animals were mated for F1 generation and continuously treated till sacrifice (12 months, chronic exposure, P generation) for blood and organ sampling. Thirty F1 mice per group were treated for 12 months before sacrifice. Schematic representation of the mice study design is depicted in Figure 5.

Fish were maintained in 50 l sterile glass tanks with 20 animals each, under standard laboratory conditions (28 ± 0.5°C, 14:10 h light: dark cycle). The water was continuously aerated, filtered and 1/3 of its volume was manually renewed every three days. Ammonia, nitrate, nitrite, pH and water hardness was monitored twice a week using commercial kit (Tetra GmbH). Fish were daily fed freshly hatched *Artemia nauplii* once and granular food (Special Diets Services) twice. Sixty 3 months old zebrafish (AB strain, 30 males and 30 females) were randomly recruited from each Ctrl, U and D treatment group, distributed in three replicate tanks for condition. After 3 months treatment, fish were sacrificed by overdose of tricaine methane sulfonate (MS222, 200-300 mg/l) by prolonged immersion and their lengths and weight recorded before organ dissection.

### Hormone measurements

Serum prepared from collected blood samples was -80°C frozen until assayed by Estradiol EIA kit (Cayman) following manufacturer’s instructions. Each sample was analyzed in triplicates. *p*-value was calculated by *t*-student test.

### RNA extraction and real-time RT-PCR

RNA was isolated from mouse and zebrafish liver tissues using Trizol reagent (Invitrogen) and purified by RNeasy mini kit (Qiagen). cDNA synthesis and qRT-PCR analysis were set for 8 animals, each carried out in triplicate as previously reported [40]. Data obtained were normalized on the relative expression of reference gene *Tubulin* in mouse and *rpl13a* in zebrafish and reported as ratio between U or D *vs* Ctrl expression values. Primer sequences are reported in Table 3. *p*-value was calculated by *t*-student test.

**Table 3:** **Genes analysed by qRT-PCR**

Gene name	Gene description	Forward primer 5’-3’	Reverse primer 5’-3’
Cdkn1a	Cyclin-dependent kinase inhibitor 1A (P21)	atccagacattcagagccacag	acgaagtcaaagttccaccgt
Hp	Haptoglobin	cttccagagagaggcaagaga	gcccaactccacagcaaaaag
Lbp	Lipopolysaccharide binding protein	gcatccagacaaggcacaag	cgaggtcgtggagctgaata
Pnrc1	Proline-rich nuclear receptor coactivator 1	ccacagacagcccccactc	tgtataccatgcacaagctggc
Ppp1r3c	Protein phosphatase 1, regulatory (inhibitor) subunit 3C	caatgagctgcaccagaatga	gtggtgaatgagccaagcaa
Sox9	SRY-box containing gene 9	tctggaggctgctgaacgag	gcttgtccgttcttcaccga
Vtn	Vitronectin	agtgcaagccccaagtaacg	ccgtccgtccgaggatttag
Nfe2l2	Nuclear factor, erythroid derived 2, like 2	gcatgatggacttggagttgc	gctcatagtccttctgtcgct
Mavs	Mitochondrial antiviral signaling protein	tatccgagacaaccacagcaa	gtcgatcaagatgactgggtg
C1qa	Complement component 1, q subcomponent, alpha polypeptide	tgtcccaccatcagcaaagg	gtctccatggtgtccctgc
C1qb	Complement component 1, q subcomponent, beta polypeptide	gacccagacttccgctttct	ctcaccccactgtgtcttca
C1qc	Complement component 1, q subcomponent, C chain	accctcaggatggtcgttgg	tgagtggtagggccagaaga
Tub-α	Tubulin-α	caacaccttcttcagtgagacagg	tacatgatctccttgccaatggt
LOC794625	Up-regulated during skeletal muscle growth protein 5	gggcaccagtttgcttgattg	cctcctgccagtgattgtgt
Cpt2	Carnitine palmitoyltransferase II	aaccgctggtacgacaa	ggacgcaggctgagaac
Rpl13a	Ribosomal protein L13A	tctggaggactgtaagaggtatgc	agacgcacaatcttgagagcag

### Microarray and bioinformatic analysis

Hepatic RNA for microarray was extracted and cleaned as reported above. Nine animals per group were randomly divided into three equivalent sets, and a constant amount of RNA from animals in the same set was pooled into one single sample in order to eliminate individual differences within group. Three arrays were used for each group. cRNA was generated by using the Affymetrix One-Cycle Target Labeling and Control Reagent kit (AffymetrixInc), following the manufacturer’s protocol, starting from 5 μg of total RNA. Biotinylated cRNA was hybridized to the GeneChip Mouse Gene 1.0 ST Array [(MoGene-1_0-st-v1, Affymetrix) and to GeneChip Zebrafish Gene 1.0 ST Arrays (Affymetrix). Chips were washed and scanned on the Affymetrix Complete GeneChip System, generating digitized image data (DAT) files.

The datasets obtained were analyzed with GeneSpring GX 12 Software (Agilent Technologies). Robust multichip average (RMA) algorithm [67, 68] was used for summarization and normalization. Hybridization quality was assessed by spiked-in controls. Principal Component Analysis (PCA) was performed to check data quality that resulted adequate for all samples. Transcripts were filtered by their signal intensity values, selecting transcripts with intensity values between 20 and 100 percentile in at least 1 out of each set samples for differential analysis.

Differentially expressed transcripts between exposed livers vs controls were filtered for absolute fold change ≥ 1.5 and corrected *p*-value ≤ 0.05. Statistical analysis was performed using Oneway ANOVA adjusted for multiple comparison by the Benjamini-Hochberg method.

Functional annotation for differentially expressed transcripts was performed using Ingenuity Pathway Analysis (IPA; http://www.ingenuity.com), a web-based tool for the identification of biological functions, canonical pathways, transcription factors as well as toxofunctions that are most significant to the dataset. Fisher Exact test was used to calculate the *p*-value determining the likelihood that the association between the set of focus genes in the dataset and a given process or pathway is due to chance alone. Corrected *p*-value calculation (based on the Benjamini-Hochberg method) controlled the error rate in analysis results and focus in on the most significant biological functions associated with DEG. Gene ontology (GO) analysis was performed using David software ( http://david.abcc.ncifcrf.gov) [69, 70]. The listed GO terms included three or more DEGs *p*-value <0.05.

### Availability of supporting data

Microarray data are available in the ArrayExpress database ( http://www.ebi.ac.uk/arrayexpress) under accession numbers E-MTAB-2905 (for mouse arrays) and E-MTAB-2906 (for zebrafish arrays).

## Electronic supplementary material

Additional file 1: **Chemical analysis of Ctrl, U and D waters.** (DOCX 14 KB)

Additional file 2: **Transcriptomic analyses in liver of acutely exposed mice.** Volcano plots of microarray data in U (A) and D (B) compared to Ctrl treated animals. The *y*-axis value is the negative logarithm base 10 of the corrected *p*-value. A green horizontal line on the plot represents the user-defined significant threshold for *p*-value. The *x*-axis is shown as the logarithm base 2 of the fold change in expression level between treated and control livers. The vertical green lines on the plot represent the user-defined thresholds for fold change. Red dots are up-regulated probes, green dots down-regulated probes. The number of down/up-regulated probes for each Volcano plot is reported in the underlying table. (TIFF 2 MB)

Additional file 3: **Zebrafish phenotypic and molecular changes induced by groundwater treatment.** (A) Body weight and (B) body length of adult zebrafish exposed for 3 months to Ctrl, U or D waters. Data are reported separately for female and male fish and, for each point, 20 animals were recorded. Volcano plots of microarray data in U (C) and D (D) compared to Ctrl treated animals. For plot description see the caption to the Additional file 1. The number of down/up-regulated probes for each Volcano plot is reported in the underlying table. (TIFF 3 MB)

Additional file 4: **Gene Ontology analysis of DEGs in acutely exposed mice.** (DOCX 19 KB)

Additional file 5: Gene Ontology analysis of DEGs in acutely exposed zebrafish. (DOCX 11 KB)

Additional file 6: **Liver molecular and phenotypic characterization of chronic exposed mice.** (A) qRT-PCR validation of selected genes (*C1qa*, *C1qb*, *C1qc*) deregulated in chronically exposed livers. Data are reported as fold change value calculated as ratio between average expression in U/D and in Ctrl exposed animals. 8 animals were analysed for each group. (B) Toxfunctions deregulated in U and D chronically exposed animal livers. Serum Urea (C), AP (D), AST (E) and ALT (F) levels measured in mice treated with Ctrl (black), U (orange) or D (blue). The analysis was conducted on 20 animals per treatment groups. Each sign is a single mouse. For each treatment group mean and standard deviation is reported. (G) Haematoxylin/eosin staining of liver sections of Ctrl, U and D chronically exposed mice. *p-value ≤0,05, **p-value ≤0,01. (TIFF 10 MB)

Additional file 7: **Material and methods.** (DOCX 19 KB)

## Abbreviations

U: Upstream water
D: Downstream water
qRT-PCR: Quantitative real time PCR
DEGs: Deregulated expressed genes
P: Parents
F1: Offspring
IPA: Ingenuity pathway analysis.
