# Supplementary material for: Cross-species toxicogenomic analyses and phenotypic anchoring in response to groundwater low-level pollution
Source: BMC Genomics. 2014 Dec 5;15(1):1067. doi: 10.1186/1471-2164-15-1067 (PMC4301944; doi:10.1186/1471-2164-15-1067)
Supplement: Supplementary file 1 — Additional file 1: Chemical analysis of Ctrl, U and D waters. (DOCX 14 KB) [file 12864_2014_6791_MOESM1_ESM.docx]

**Additional file 1** Chemical analysis of Ctrl, U and D waters.

| Element/  compound | T-Water µg/L | L-Water µg/L | C-Water µg/L |
| --- | --- | --- | --- |
| Lithium | 100.387 | 286.117 | 9.107 |
| Beryllium | 0.025 | 0.012 | 0.110 |
| Aluminium | <0.000 | <0.000 | 4.589 |
| Chromium | 4.742 | 3.638 | 1.231 |
| Manganese | 418.880 | 131.718 | 0.083 |
| Cobalt | 0.677 | 0.163 | 0.041 |
| Nickel | 20.120 | 4.596 | 0.459 |
| Cupper | 8.300 | 3.195 | 0.727 |
| Zinc | 70.447 | 70.3 | 29.253 |
| Gallium | 0.066 | 0.054 | 7.575 |
| Arsenic | 0.011 | <0.000 | 5.620 |
| Palladium | <0.000 | <0.000 | 1.215 |
| Cadmium | 0.021 | 0.015 | 0.011 |
| Stannum | <0.000 | <0.000 | 0.059 |
| Stibium | 0.298 | 0.201 | 0.209 |
| Barium | 0.063 | 0.053 | 0.136 |
| Lanthaum | <0.000 | <0.000 | <0.000 |
| Hafnium | <0.000 | <0.000 | 0.100 |
| Tantalum | <0.000 | <0.000 | 0.013 |
| Tungsten | <0.000 | <0.000 | 0.123 |
| Thallium | <0.000 | <0.000 | 0.026 |
| Lead | 0.128 | <0.000 | <0.000 |
| Tetraethyl silicate | 0.14 | 0.15 | <0.000 |
| 2,4-dimethylphenol | 0.29 | 0.11 | <0.000 |
| Dibutyl phthalate | 4.01 | 5.11 | <0.000 |
| Diisobutyl phthalate | 0.44 | 0.55 | <0.000 |
| Squalene | 0.54 | 0.45 | <0.000 |
| Chloroform | 0.68 | 0.78 | <0.000 |
| Toluene | 0.48 | 0.53 | <0.000 |
| O-Xilene | 0.21 | <0.000 | <0.000 |
| dichloromethane | 0.24 | 0.34 | <0.000 |
| chloroethane | 0.23 | 0.23 | <0.000 |
| dibromoethane | 0.14 |  | <0.000 |
| Phenol | 0.08 | 0.06 | <0.000 |
| phthalic acid | 0.03 | 0.07 | <0.000 |
| 2,4-Di-tert-butylphenol | 21.12 | 13.58 | <0.000 |
| di-n-octylphthalate | 29.85 | 17.76 | <0.000 |
