# Supplementary figures and images for: Cross-species toxicogenomic analyses and phenotypic anchoring in response to groundwater low-level pollution
Source: BMC Genomics. 2014 Dec 5;15(1):1067. doi: 10.1186/1471-2164-15-1067 (PMC4301944; doi:10.1186/1471-2164-15-1067)

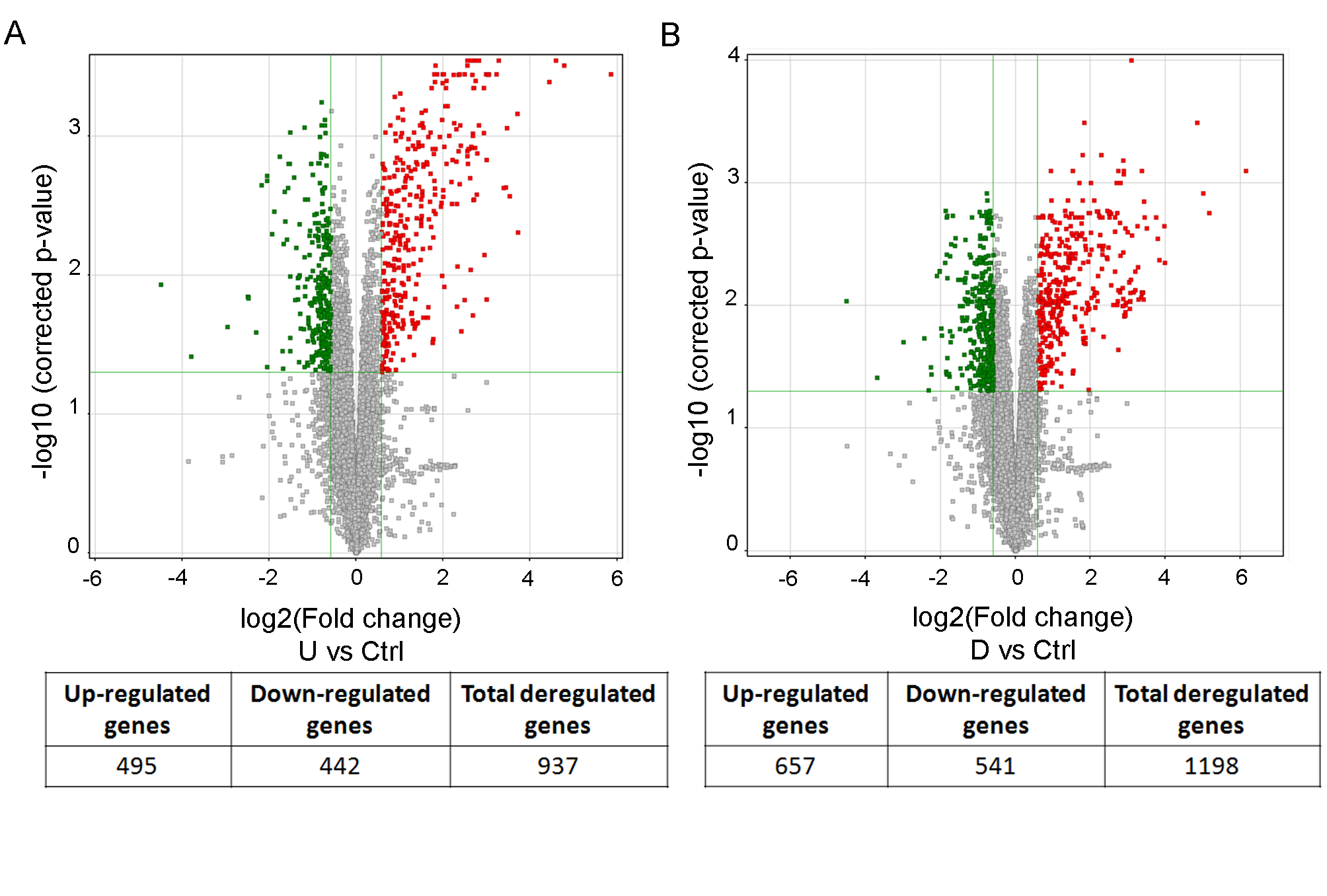

Supplement: Supplementary file 2 — Additional file 2: Transcriptomic analyses in liver of acutely exposed mice. Volcano plots of microarray data in U (A) and D (B) compared to Ctrl treated animals. The y-axis value is the negative logarithm base 10 of the corrected p-value. A green horizontal line on the plot represents the user-defined significant threshold for p-value. The x-axis is shown as the logarithm base 2 of the fold change in expression level between treated and control livers. The vertical green lines on the plot represent the user-defined thresholds for fold change. Red dots are up-regulated probes, green dots down-regulated probes. The number of down/up-regulated probes for each Volcano plot is reported in the underlying table. (TIFF 2 MB) [file 12864_2014_6791_MOESM2_ESM.tiff]

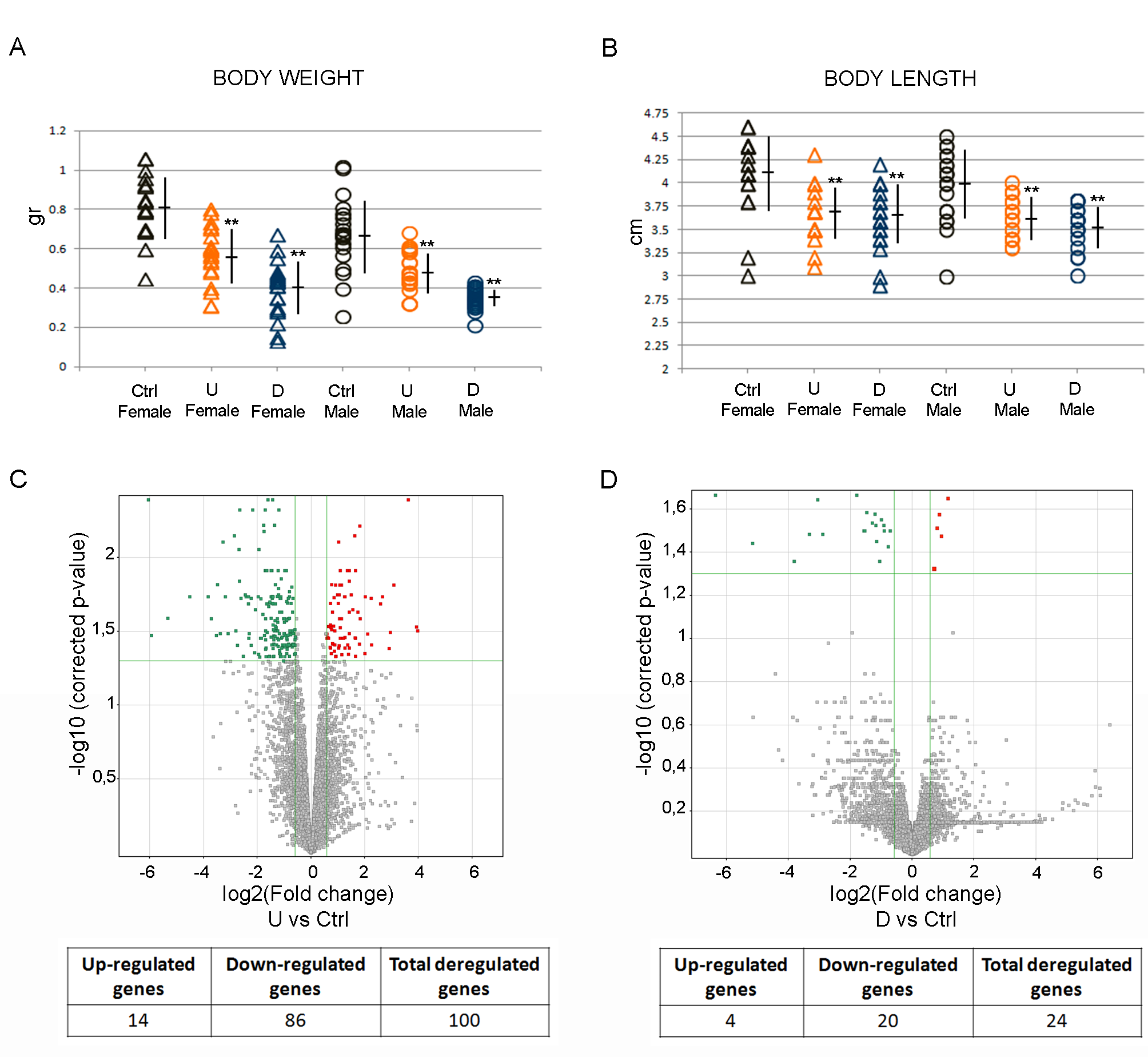

Supplement: Supplementary file 3 — Additional file 3: Zebrafish phenotypic and molecular changes induced by groundwater treatment. (A) Body weight and (B) body length of adult zebrafish exposed for 3 months to Ctrl, U or D waters. Data are reported separately for female and male fish and, for each point, 20 animals were recorded. Volcano plots of microarray data in U (C) and D (D) compared to Ctrl treated animals. For plot description see the caption to the Additional file 1. The number of down/up-regulated probes for each Volcano plot is reported in the underlying table. (TIFF 3 MB) [file 12864_2014_6791_MOESM3_ESM.tiff]

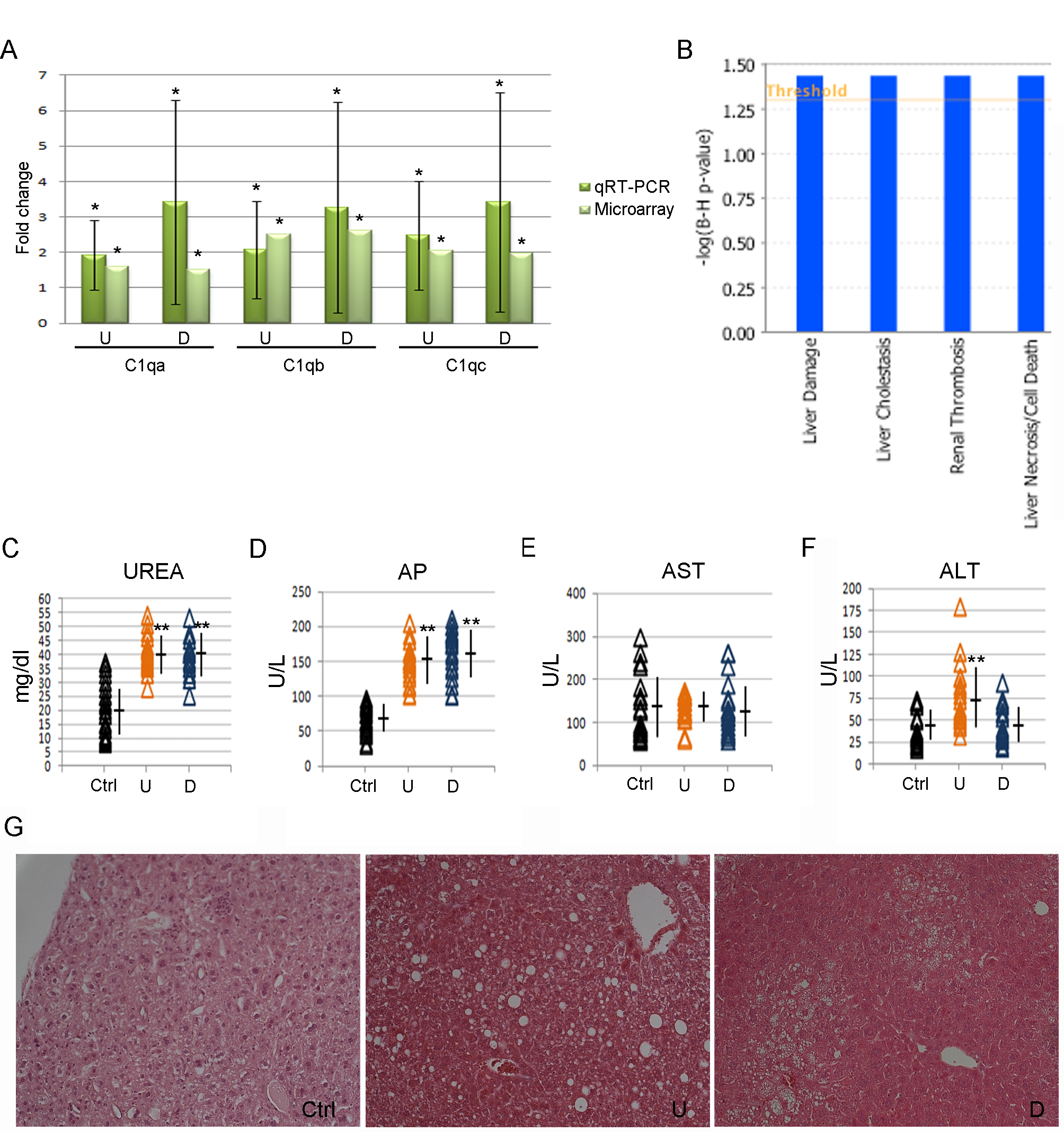

Supplement: Supplementary file 6 — Additional file 6: Liver molecular and phenotypic characterization of chronic exposed mice. (A) qRT-PCR validation of selected genes (C1qa, C1qb, C1qc) deregulated in chronically exposed livers. Data are reported as fold change value calculated as ratio between average expression in U/D and in Ctrl exposed animals. 8 animals were analysed for each group. (B) Toxfunctions deregulated in U and D chronically exposed animal livers. Serum Urea (C), AP (D), AST (E) and ALT (F) levels measured in mice treated with Ctrl (black), U (orange) or D (blue). The analysis was conducted on 20 animals per treatment groups. Each sign is a single mouse. For each treatment group mean and standard deviation is reported. (G) Haematoxylin/eosin staining of liver sections of Ctrl, U and D chronically exposed mice. *p-value ≤0,05, **p-value ≤0,01. (TIFF 10 MB) [file 12864_2014_6791_MOESM6_ESM.tiff]
