# Supplementary material for: Cross-species toxicogenomic analyses and phenotypic anchoring in response to groundwater low-level pollution
Source: BMC Genomics. 2014 Dec 5;15(1):1067. doi: 10.1186/1471-2164-15-1067 (PMC4301944; doi:10.1186/1471-2164-15-1067)
Supplement: Supplementary file 4 — Additional file 4: Gene Ontology analysis of DEGs in acutely exposed mice. (DOCX 19 KB) [file 12864_2014_6791_MOESM4_ESM.docx]

**Additional file 4** Gene Ontology analysis of DEGs in acutely exposed mice

| **Category** | **GO Term** | **PValue** |
| --- | --- | --- |
| GOTERM_CC | ribosome | 2.80E-25 |
| GOTERM_MF | structural constituent of ribosome | 1.21E-24 |
| GOTERM_BP | translation | 2.39E-19 |
| GOTERM_CC | ribonucleoprotein complex | 3.53E-19 |
| GOTERM_MF | structural molecule activity | 1.65E-15 |
| GOTERM_CC | mitochondrion | 1.71E-08 |
| GOTERM_CC | intracellular non-membrane-bounded organelle | 1.72E-08 |
| GOTERM_CC | non-membrane-bounded organelle | 1.72E-08 |
| GOTERM_MF | rRNA binding | 3.43E-08 |
| GOTERM_CC | ribosomal subunit | 7.01E-08 |
| GOTERM_CC | respiratory chain | 7.12E-07 |
| GOTERM_BP | electron transport chain | 3.30E-05 |
| GOTERM_CC | small ribosomal subunit | 7.86E-05 |
| GOTERM_BP | generation of precursor metabolites and energy | 1.01E-04 |
| GOTERM_BP | ATP synthesis coupled electron transport | 1.07E-04 |
| GOTERM_CC | cytosolic ribosome | 1.51E-04 |
| GOTERM_CC | large ribosomal subunit | 3.36E-04 |
| GOTERM_CC | mitochondrial ribosome | 4.26E-04 |
| GOTERM_CC | organellar ribosome | 4.26E-04 |
| GOTERM_CC | mitochondrial part | 5.81E-04 |
| GOTERM_BP | respiratory electron transport chain | 6.43E-04 |
| GOTERM_MF | RNA binding | 7.69E-04 |
| GOTERM_BP | innate immune response | 8.76E-04 |
| GOTERM_BP | mitochondrial ATP synthesis coupled electron transport | 9.43E-04 |
| GOTERM_BP | oxidative phosphorylation | 1.22E-03 |
| GOTERM_CC | mitochondrial inner membrane | 2.46E-03 |
| GOTERM_CC | cytosolic small ribosomal subunit | 3.52E-03 |
| GOTERM_CC | organelle inner membrane | 3.74E-03 |
| GOTERM_MF | NADH dehydrogenase (ubiquinone) activity | 4.88E-03 |
| GOTERM_MF | NADH dehydrogenase activity | 4.88E-03 |
| GOTERM_MF | NADH dehydrogenase (quinone) activity | 4.88E-03 |
| GOTERM_CC | mitochondrial membrane | 5.28E-03 |
| GOTERM_CC | mitochondrial large ribosomal subunit | 5.33E-03 |
| GOTERM_CC | organellar large ribosomal subunit | 5.33E-03 |
| GOTERM_BP | acute inflammatory response | 6.14E-03 |
| GOTERM_MF | hydrogen ion transmembrane transporter activity | 6.79E-03 |
| GOTERM_MF | oxidoreductase activity, acting on NADH or NADPH, quinone or similar compound as acceptor | 6.83E-03 |
| GOTERM_BP | immune response | 8.06E-03 |
| GOTERM_MF | monovalent inorganic cation transmembrane transporter activity | 8.68E-03 |
| GOTERM_CC | mitochondrial envelope | 8.69E-03 |
| GOTERM_CC | membrane-enclosed lumen | 9.20E-03 |
| GOTERM_BP | cellular respiration | 1.01E-02 |
| GOTERM_MF | inorganic cation transmembrane transporter activity | 1.05E-02 |
| GOTERM_CC | organelle lumen | 1.08E-02 |
| GOTERM_BP | defense response | 1.27E-02 |
| GOTERM_BP | energy derivation by oxidation of organic compounds | 1.34E-02 |
| GOTERM_BP | humoral immune response mediated by circulating immunoglobulin | 1.36E-02 |
| GOTERM_BP | inflammatory response | 1.60E-02 |
| GOTERM_CC | nucleosome | 1.61E-02 |
| GOTERM_CC | intracellular organelle lumen | 1.83E-02 |
| GOTERM_CC | polysome | 1.97E-02 |
| GOTERM_BP | nucleosome assembly | 2.08E-02 |
| GOTERM_BP | ribosome biogenesis | 2.26E-02 |
| GOTERM_BP | chromatin assembly | 2.27E-02 |
| GOTERM_CC | organelle envelope | 2.28E-02 |
| GOTERM_CC | envelope | 2.35E-02 |
| GOTERM_BP | lymphocyte mediated immunity | 2.37E-02 |
| GOTERM_BP | nucleosome organization | 2.37E-02 |
| GOTERM_BP | protein-DNA complex assembly | 2.37E-02 |
| GOTERM_CC | small nucleolar ribonucleoprotein complex | 2.54E-02 |
| GOTERM_BP | response to bacterium | 2.59E-02 |
| GOTERM_BP | response to wounding | 2.83E-02 |
| GOTERM_CC | protein-DNA complex | 3.00E-02 |
| GOTERM_BP | oxidation reduction | 3.26E-02 |
| GOTERM_BP | immune effector process | 3.51E-02 |
| GOTERM_BP | activation of immune response | 3.51E-02 |
| GOTERM_BP | erythrocyte homeostasis | 3.66E-02 |
| GOTERM_BP | positive regulation of transcription, DNA-dependent | 3.77E-02 |
| GOTERM_MF | oxidoreductase activity, acting on NADH or NADPH | 3.78E-02 |
| GOTERM_BP | chromatin organization | 3.79E-02 |
| GOTERM_BP | leukocyte mediated immunity | 3.91E-02 |
| GOTERM_BP | positive regulation of RNA metabolic process | 3.94E-02 |
| GOTERM_BP | humoral immune response | 4.23E-02 |
| GOTERM_MF | oxidoreductase activity, acting on heme group of donors | 4.35E-02 |
| GOTERM_MF | oxidoreductase activity, acting on heme group of donors, oxygen as acceptor | 4.35E-02 |
| GOTERM_MF | cytochrome-c oxidase activity | 4.35E-02 |
| GOTERM_MF | heme-copper terminal oxidase activity | 4.35E-02 |
| GOTERM_BP | positive regulation of immune response | 4.63E-02 |
| GOTERM_BP | ribonucleoprotein complex biogenesis | 4.75E-02 |
| GOTERM_BP | regulation of cellular protein metabolic process | 4.92E-02 |
| GOTERM_BP | myeloid cell differentiation | 4.93E-02 |
|  |  |  |
|  |  |  |
