# Supplementary material for: Cross-species toxicogenomic analyses and phenotypic anchoring in response to groundwater low-level pollution
Source: BMC Genomics. 2014 Dec 5;15(1):1067. doi: 10.1186/1471-2164-15-1067 (PMC4301944; doi:10.1186/1471-2164-15-1067)
Supplement: Supplementary file 5 — Additional file 5: Gene Ontology analysis of DEGs in acutely exposed zebrafish. (DOCX 11 KB) [file 12864_2014_6791_MOESM5_ESM.docx]

**Additional file 5** Gene Ontology analysis of DEGs in acutely exposed zebrafish

| **Category** | **GO Term** | **PValue** |
| --- | --- | --- |
| GOTERM_BP | protein folding | 6.79E-03 |
| GOTERM_CC | endoplasmic reticulum | 4.05E-02 |
| GOTERM_CC | mitochondrion | 4.88E-02 |
