# Supplementary material for: Cross-species toxicogenomic analyses and phenotypic anchoring in response to groundwater low-level pollution
Source: BMC Genomics. 2014 Dec 5;15(1):1067. doi: 10.1186/1471-2164-15-1067 (PMC4301944; doi:10.1186/1471-2164-15-1067)
Supplement: Supplementary file 7 — Additional file 7: Material and methods. (DOCX 19 KB) [file 12864_2014_6791_MOESM7_ESM.docx]

**Supplementary material**

**Material and methods**

**Inductively Coupled Plasma Mass Spectrometry (ICP-MS) analysis**

Aliquots of water solution (1ml) from each sample were directly analyzed by ICP-MS. Samples were transferred into polystyrene liners and diluted 1:10 v/v with 5% HNO_3_ before the analyses with an Agilent 7700 ICP-MS from Agilent Technologies, equipped with a frequency-matching RF generator and 3rd generation Octopole Reaction System (ORS3), operating with helium gas in ORF. The following parameters were used: radiofrequency power 1550 W, plasma gas flow 14 l/min; carrier gas flow 0.99 l/min; He gas flow 4.3 ml/min. 103Rh was used as an internal standard (50 μg/l final concentration). Multi-element calibration standards were prepared in 5% HNO_3_ at 4 different concentrations (1, 10, 50, and 100 μg/l).

**Gas chromatography Mass Spectrometry (GC-MS) analysis**

Water aliquots were submitted to liquid-liquid extraction procedure by using an equal amount of chloroform, hexane and dichloromethane (1:1 v/v). The extraction step was performed three times and the organic substances were collected and dried under nitrogen and dissolved in 200 μl of hexane. The hexane supernatant (1/200) was used for the GC-MS analysis performed on a 5390 MSD quadrupole mass spectrometer (Agilent Technologies) equipped with a gas chromatograph by using a SPB-5 fused silica capillary column (30 m, 0.5 mm ID, 0.25 μm ft) from Supelco. The injection temperature was 250°C. The oven temperature was increased from 40°C to 90°C in 1 min and held at 90°C for 1 min before increasing to 140°C at 25°C/min, to 200°C at 5°C/min and finally to 300°C at 10°C/min. Electron Ionisation (EI) mass spectra were recorded by continuous quadrupole scanning at 70eV ionisation energy.

**Histopathological analysis**

Mouse liver tissues fixed in 10% formalin were processed sequentially in ethanol, xylene and paraffin. Tissues were embedded in paraffin wax, sectioned (4-5µm), and mounted on slides. The sections were subsequently stained with haematoxylin and eosin**.**
